# Implication of PMLIV in Both Intrinsic and Innate Immunity

**DOI:** 10.1371/journal.ppat.1003975

**Published:** 2014-02-27

**Authors:** Faten El Asmi, Mohamed Ali Maroui, Jacques Dutrieux, Danielle Blondel, Sébastien Nisole, Mounira K. Chelbi-Alix

**Affiliations:** 1 INSERM UMR-S 1124, Paris, France; 2 Université Paris Descartes, Paris, France; 3 CNRS UPR 3296, Gif sur Yvette, France; Harvard Medical School, United States of America

## Abstract

PML/TRIM19, the organizer of nuclear bodies (NBs), has been implicated in the antiviral response to diverse RNA and DNA viruses. Several PML isoforms generated from a single *PML* gene by alternative splicing, share the same N-terminal region containing the RBCC/tripartite motif but differ in their C-terminal sequences. Recent studies of all the PML isoforms reveal the specific functions of each. The knockout of *PML* renders mice more sensitive to vesicular stomatitis virus (VSV). Here we report that among PML isoforms (PMLI to PMLVIIb), only PMLIII and PMLIV confer resistance to VSV. Unlike PMLIII, whose anti-VSV activity is IFN-independent, PMLIV can act at two stages: it confers viral resistance directly in an IFN-independent manner and also specifically enhances IFN-β production *via* a higher activation of IRF3, thus protecting yet uninfected cells from oncoming infection. PMLIV SUMOylation is required for both activities. This demonstrates for the first time that PMLIV is implicated in innate immune response through enhanced IFN-β synthesis. Depletion of IRF3 further demonstrates the dual activity of PMLIV, since it abrogated PMLIV-induced IFN synthesis but not PMLIV-induced inhibition of viral proteins. Mechanistically, PMLIV enhances IFN-β synthesis by regulating the cellular distribution of Pin1 (peptidyl-prolyl cis/trans isomerase), inducing its recruitment to PML NBs where both proteins colocalize. The interaction of SUMOylated PMLIV with endogenous Pin1 and its recruitment within PML NBs prevents the degradation of activated IRF3, and thus potentiates IRF3-dependent production of IFN-β. Whereas the intrinsic antiviral activity of PMLIV is specific to VSV, its effect on IFN-β synthesis is much broader, since it affects a key actor of innate immune pathways. Our results show that, in addition to its intrinsic anti-VSV activity, PMLIV positively regulates IFN-β synthesis in response to different inducers, thus adding PML/TRIM19 to the growing list of TRIM proteins implicated in both intrinsic and innate immunity.

## Author Summary

PML is expressed as seven isoforms, designated PMLI to PMLVIIb, each with specific functions conferred by their C-terminal regions. PML isoforms are implicated in several cell processes, including antiviral defense. Very few studies have been performed with all PML isoforms to investigate their individual antiviral properties. Our comparative study with all PML isoforms on VSV replication revealed that only PMLIII and PMLIV are able to inhibit this virus in an IFN-independent way. Importantly, PMLIV is also able to enhance IRF3 phosphorylation resulting in a dramatic IFN-β production in response to viral infection, thus protecting yet uninfected cells. At the opposite, the specific suppression of PMLIV expression in human cells reduced virus-induced IRF3 activation and subsequent IFN-β production. We found that PMLIV affects the endogenous distribution of Pin1, by recruiting this protein in PML NBs where both proteins colocalize. This prevents activated IRF3 degradation, thus enhancing the production of IFN-β. Here we show for the first time that, in addition to its intrinsic anti-VSV activity, PMLIV is implicated in antiviral innate immune response. Thus, *PML* is an IFN stimulated gene whose products have a broad intrinsic antiviral activity and one of them, PMLIV, is also a potent regulator of innate immune pathways.

## Introduction

The establishment of an antiviral state in cells is the defining property of interferons (IFNs), as well as the activity that led to their discovery. IFNs are the first line of defense against viral infections. IFN-regulatory factor 3 (IRF3), a ubiquitously expressed transcription factor, is responsible for the primary induction of IFN and is a crucial player in the establishment of innate immunity in response to viral infection [1]. IFNs bind to their receptors and activate the canonical JAK/STAT pathway, leading to the induction of IFN-stimulated genes (ISGs), whose products mediate their biological effects [2], [3].

Vesicular Stomatitis Virus (VSV) belongs to the *Rhabdoviridae* family. Its single stranded negative sense RNA genome (about 12 kb), that encodes 5 viral proteins, is encapsidated by the nucleoprotein N to form the nucleocapsid that is associated with the RNA-dependent RNA polymerase L and its cofactor the phosphoprotein P. Inside the viral particle, this nucleocapsid is associated with the matrix protein M and surrounded by a membrane containing a unique glycoprotein G. The virus enters the host cell through the endosomal transport pathway *via* a low-pH-induced membrane fusion process catalyzed by the glycoprotein G [4]. The nucleocapsid released into the cytoplasm serves as a template for transcription and replication processes that are catalyzed by the L-P polymerase complex [5], [6]. During transcription, a positive–stranded leader RNA and five capped and polyadenylated mRNAs are synthesized. These viral mRNA are translated by the cellular machinery to give the viral proteins N, P, M, G and L, then the replication process yields full-length antigenome-sense RNA, which in turn serve as templates for the synthesis of genome-sense RNA. During their synthesis, both the nascent antigenome and the genome are encapsidated by N proteins. The neo-synthesized genome either serves as a template for secondary transcription or is assembled with M proteins to allow budding of the neosynthesized virion at a cellular membrane.

VSV replication is highly sensitive to the inhibitory action of IFN and is routinely used to assay the antiviral activity of IFN *in vitro* [7]. Although IFN treatment induces the expression of hundreds of ISGs, only a few of them have been demonstrated to be responsible for the inhibition of VSV replication. Indeed, ISG products such as double-stranded RNA-activated protein kinase (PKR) [8], myxovirus resistance protein (Mx) [9], p53 [10], ISG20 [11], *Ifit2*/ISG54 [12] and ProMyelocytic Leukemia (PML) [13], [14] have been reported to confer resistance to VSV infection. In addition, 34 ISG products including PML have been shown to elicit an antiviral effect on VSV replication [15].

PML (also named TRIM19 for TRIpartite Motif protein 19) is the organizer of small nuclear-matrix structures named nuclear bodies (NBs) [16]. In response to diverse stimuli, PML NBs recruit a growing number of proteins implicated in different cellular processes such as DNA damage response, apoptosis, senescence, protein degradation and antiviral defense [17]–[25]. PML is covalently conjugated to small ubiquitin modifier (SUMO) on three major lysine residues (K65, K160, K490) [26]. This modification, which affects PML localization, stability and ability to interact with other partners, is critical for NB functions [16], [27].

Several PML isoforms generated by alternative splicing from a single gene are designated PMLI to PMLVIIb [28], [29]. They share the same N-terminal region, which encodes the RBCC/TRIM (RING finger, B-box, and Coiled-Coil) motif, but differ in their C-terminal region due to alternative splicing. The variability of the C-terminal part of PML isoforms is important for the recruitment of specific interacting partners and for the specific function of each [29]. The implication of PML in antiviral defense against RNA and DNA viruses from different families has been demonstrated in cells stably expressing individual PML isoform or in cells depleted for PML by RNA interference (reviewed in [17], [21]). We have previously shown that PMLIII confers resistance to VSV [13]. The antiviral effect of PML has been observed *in vivo*, as PML deficiency renders mice more susceptible to VSV infection [14]. The role of the other PML isoforms in VSV-infected cells is so far unknown. Therefore, we studied the implication of all PML isoforms during VSV infection. We show that only stable expression of PMLIII or PMLIV conferred resistance to VSV infection. Whereas the activity of PMLIII was saturated at a high multiplicity of infection (MOI), PMLIV conferred a higher protective effect towards VSV infection, even at high MOIs. Finally, unlike PMLIII whose anti-VSV activity is strictly IFN-independent, we show that PMLIV confers a strong resistance to VSV infection *via* two independent mechanisms. Indeed, PMLIV is able to block viral replication in an IFN-independent manner, and also to trigger innate immunity pathways, leading to higher activation of IRF3 and specific enhancement of IFN-β production. Both activities of PMLIV required its SUMOylation. The peptidyl-prolyl isomerase (Pin1) that is known to interact with phosphorylated IRF3 and to promote its degradation *via* the ubiquitin-proteasome pathway [30], was recruited within PML NBs in human cells expressing PMLIV. Therefore, the interaction of endogenous Pin1 with SUMOylated PMLIV and its recruitment within NBs resulted in an enhanced IRF3-dependent production of IFN-β in response to VSV infection and also to other inducers such as Sendai virus (SeV), encephalomyocarditis virus (EMCV), human T-lymphotropic virus type 1 (HTLV-1), influenza virus, vaccinia virus or poly(I:C). Remarkably, specific depletion of PMLIV in human cells reduced both SeV-induced IRF3 activation and IFN-β production. Our results demonstrate for the first time PMLIV's involvement in both intrinsic and innate immunity.

## Results

### PMLIII and PMLIV inhibit VSV replication

**Figure 1:**
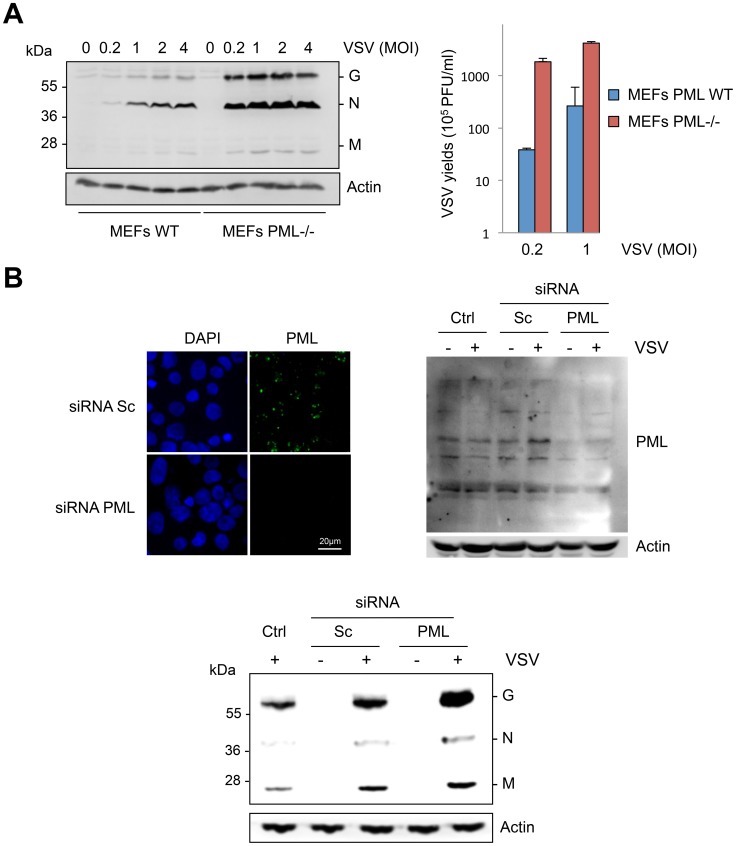
Cells are more susceptible to VSV infection in the absence of PML. (A) MEFs WT and MEFs PML-/- were infected with VSV at different MOIs for 8 h. Cell extracts were analyzed by Western blot and revealed by anti-VSV and anti-Actin antibodies (left panel) and the supernatants from cells infected at MOI of 0.2 or 1 were used for the determination of virus yields (right panel). Means and standard deviations of two independent experiments are shown. (B) Human U373MG cells control (Ctrl), transfected with siRNA scramble (Sc) or siRNA specific to PML were infected with VSV at an MOI of 0.2 for 8 h. Immunofluorescence analysis was performed using anti-PML antibody (left panel) and the cell extracts were analyzed by Western blot and revealed by anti-PML (right panel), anti-VSV (bottom panel) and anti-Actin antibodies.

As *PML* knockout mice are more sensitive to VSV infection than parental mice [14], we studied viral protein expression and viral production in MEFs derived from these mice. The five structural proteins of VSV are the nucleocapsid, N, the matrix protein, M, the glycoprotein, G, and two minor proteins, the phosphoprotein, P, and the polymerase protein, L, but only the G, N, and M proteins were revealed with our rabbit anti-VSV antibodies. VSV proteins and VSV production were lower in MEFs WT compared to MEFs PML-/- (Figure 1A). In addition, down-regulation of PML expression by RNA interference in human U373MG cells boosted VSV protein expression (Figure 1B).

**Figure 2:**
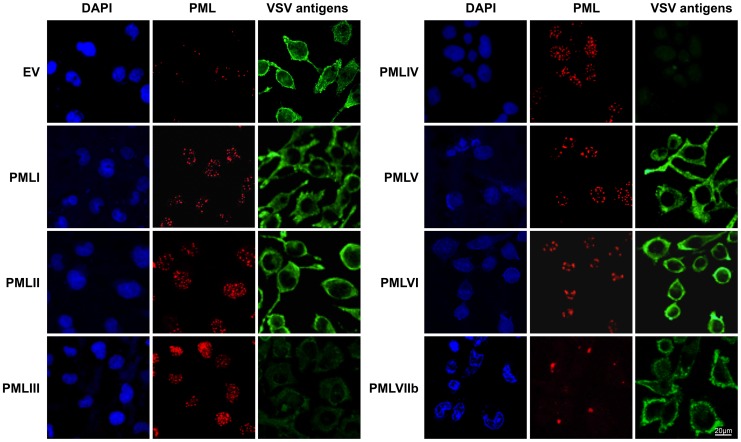
Immunofluorescence analysis on infected cells expressing each PML isoform. U373MG cells transfected with the empty vector (EV) or U373MG cells stably expressing PMLI, PMLII, PMLIII, PMLIV, PMLV, PMLVI, or PMLVIIb were infected with VSV at an MOI of 0.1 for 12 h. Double immunofluorescence analyses were performed using monoclonal anti-PML (red) and rabbit anti-VSV (green) antibodies.

**Figure 3:**
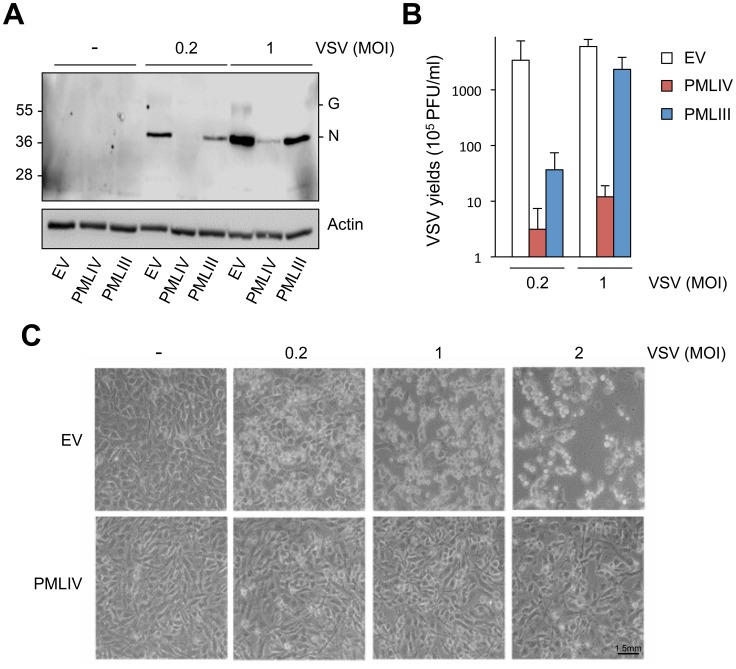
Compared to PMLIII, PMLIV has a higher anti-VSV activity. (A) U373MG-EV, U373MG-PMLIII or U373MG-PMLIV cells were not infected (-) or infected with VSV at MOIs of 0.2 or 1 for 8 h. Cell extracts were analyzed by Western blotting and revealed by anti-VSV and anti-Actin antibodies. (B) Supernatants from infected U373MG-EV, U373MG-PMLIII or U373MG-PMLIV cells were used for the determination of the virus yields. Mean values and standard deviations of three independent experiments are shown. (C) PMLIV protected cells from VSV-induced cell lysis. U373MG-EV and U373MG-PMLIV cells were left uninfected (-) or infected with VSV at MOIs of 0.2, 1 or 2 for 12 h. The phase contrast picture was acquired using a Nikon Eclipse TS100 microscope and Coolpix MDC lens camera.

To determine which PML isoforms were capable of inhibiting VSV replication, we infected at an MOI of 0.1 U373MG cells transfected with an empty vector (EV) or stably expressing each PML isoform (PMLI, PMLII, PMLIII, PMLIV, PMLV, PMLVI, or PMLVIIb). The expression level of the different PML isoforms is shown in Figure S1A. Double immunofluorescence staining for PML and VSV antigens revealed that only PMLIII and PMLIV inhibited expression of viral proteins (Figure 2). Western blot of extracts from all these cells infected at different MOIs confirmed these results (Figure S1B). As previously shown [13], the capacity of PMLIII to inhibit viral protein expression was observed at low MOIs but decreased at higher MOI, whereas PMLIV inhibited viral protein synthesis even at an MOI of 2 (Figure S1B). To further confirm this result, extracts from U373MG-EV, U373MG-PMLIII and U373MG-PMLIV cells infected at MOIs of 0.2 or 1 were analyzed in the same Western blot and their supernatants were used for the determination of virus yield by standard plaque assay. As seen in Figure 3, compared to PMLIII, PMLIV had a higher capacity to inhibit viral protein synthesis and VSV multiplication. Compared to control cells, PMLIII had a slight effect on VSV production (2-fold inhibition) when cells were infected at an MOI of 1 for 8 h, whereas PMLIV exhibited a 500-fold inhibition (Figure 3B). Furthermore, compared to control EV cells, U373MG-PMLIV cells were protected against cell lysis 12 h post-infection at different MOIs (Figure 3C), whereas U373MG-PMLIII cells were protected only at a low MOI of 0.2 (data not shown). To further confirm the effect of PMLIV on VSV infection, we tested another cell clone stably expressing PMLIVa, a PMLIV variant, lacking exon 5 but having the same C-terminal region. This PML isoform was also able to inhibit VSV protein expression, as observed with PMLIV (data not shown).

Taken together, these results demonstrate that among all PML isoforms, only PMLIII, PMLIV and PMLIVa conferred resistance to VSV. However, PMLIV and PMLIVa mediated a much higher protection, suggesting the crucial role of their specific C-terminal portion.

### PMLIV does not alter VSV entry but inhibits the secondary viral transcription

**Figure 4:**
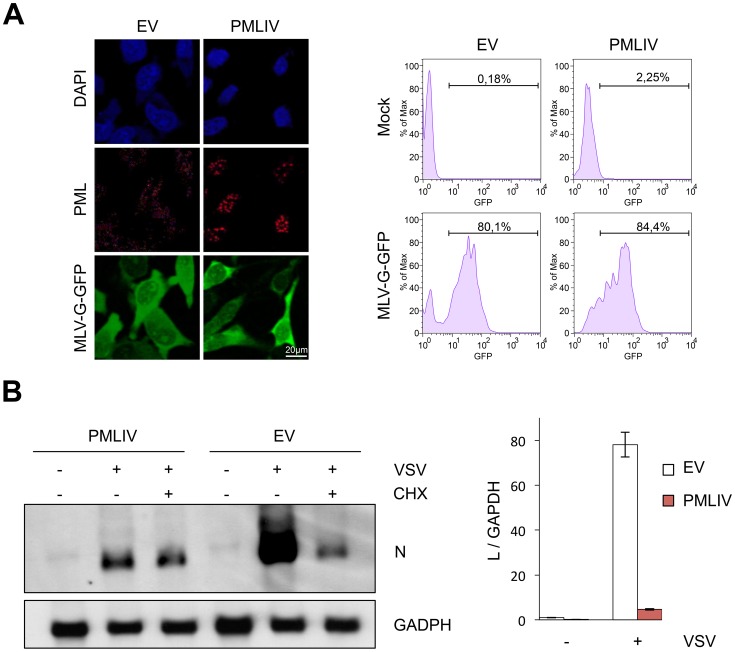
PMLIV does not alter VSV entry and inhibits viral transcription. (A) U373MG-EV and U373MG-PMLIV cells were infected with MLV-GFP/G for 48 h. Double staining of PML and GFP was presented (left panel) and the transduction efficiency, expressed as the percentage of GFP-positive cells, was measured 48 h post-infection using a fluorescence-activated cell sorter (right panel). (B) PMLIV inhibited viral mRNA transcription. U373MG-EV and U373MG-PMLIV cells were infected by VSV at an MOI of 3 in the absence (−CHX) or the presence (+CHX) of cycloheximide. After 4 h of infection, samples were analyzed for the presence of VSV-N and GAPDH mRNAs (left panel). Total RNA were used for VSV-L mRNA quantification by RT-qPCR (right panel). The values obtained for uninfected cells expressing the empty vector (EV) were arbitrarily set to 1. Means and standard deviations of three independent experiments are shown.

To investigate which viral step is targeted by PMLIV, we first investigated whether VSV entry was affected. Thus, we used a MLV virus encoding GFP pseudotyped with the receptor-binding G protein (VSV-G) [31]. This pseudovirus can undergo VSV-G-mediated entry but cannot produce its own VSV-G envelope, and hence is only capable of a single-round of infection. MLV-G-GFP was used to transduce U373MG-EV and U373MG-PMLIV cells and GFP expression was readily detected 48 h later by immunofluorescence (Figure 4A, left panel) and flow cytometry (Figure 4A, right panel). These analyses revealed that U373MG-EV and U373MG-PMLIV cells had similar GFP staining reaching 80.1% and 84.4% GFP^+^ cells, respectively. These results demonstrate that VSV entry was not affected in PMLIV expressing cells.

Next, to determine whether viral transcription was altered by PMLIV expression, U373MG-EV and U373MG-PMLIV cells were left uninfected or infected with VSV at an MOI of 3 for 4 h. Total RNA from cell extracts was analyzed by Northern blot for VSV-N mRNA (Figure 4B, left panel) and VSV-L mRNA quantified by RT-qPCR (Figure 4B, right panel). Compared to U373MG-EV cells, the amount of N and L mRNAs was highly reduced in U373MG-PMLIV cells (Figure 4B). It is known that treatment of cells with the protein synthesis inhibitor cycloheximide (CHX) results exclusively in primary mRNA synthesis, as viral genome replication requires the ongoing synthesis of N protein [32]. Interestingly, the N mRNA level was comparable to the level synthesized in the presence of CHX, indicating that secondary transcription was inhibited by the PMLIV expression (Figure 4B, left panel) whereas primary transcription was not. These data suggest that PMLIV had no effect on steps preceding transcription but restricts a post-transcriptional step involving protein synthesis and replication. It is therefore possible that PMLIV induces IFN synthesis during VSV infection, which may in turn inhibit viral mRNA and protein expression.

### PMLIV enhances IFN-β synthesis without affecting that of IFN-α, TNF-α or IL8

**Figure 5:**
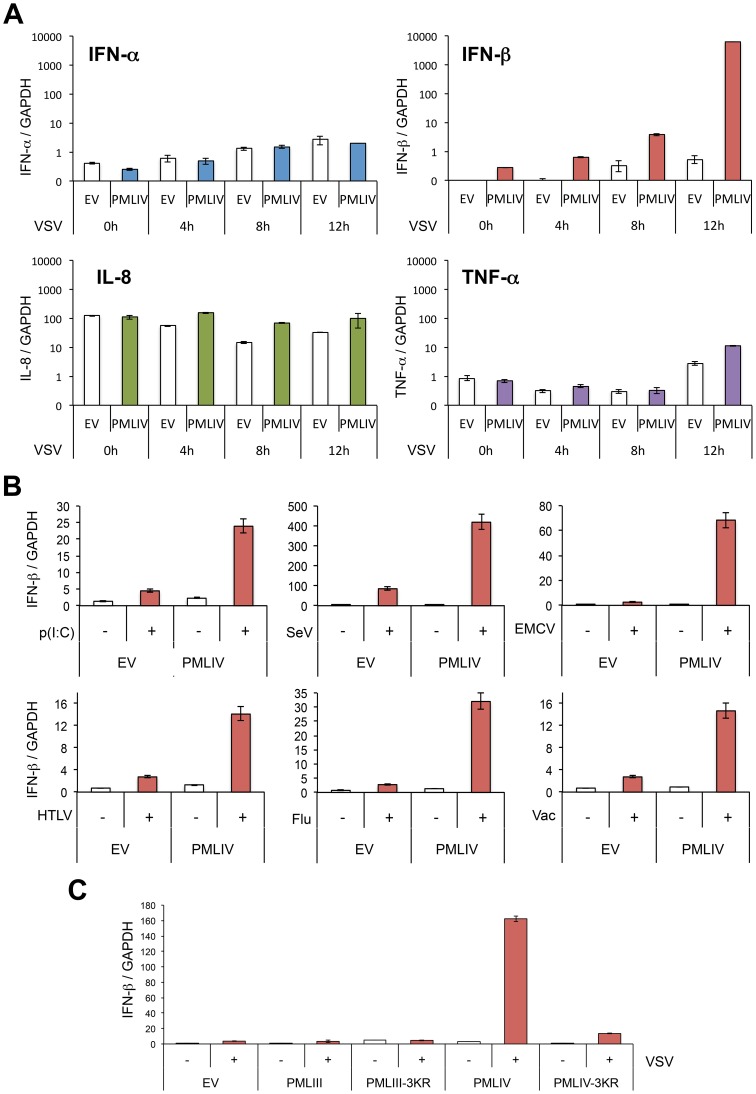
PMLIV positively regulates IFN-β synthesis. (A) PMLIV enhances IFN-β synthesis without affecting that of IFN-α, IL8 or TNF-α upon VSV infection. U373MG-EV and U373MG-PMLIV cells were infected with VSV at an MOI of 0.2 for different lengths of time. Total RNA was extracted and mRNAs encoding IFN-α, IFN-β, IL8, TNF-α, and GAPDH were quantified by RT-qPCR. (B) PMLIV positively regulates IFN-β synthesis in cells infected with SeV or transfected with poly(I:C). U373MG-EV and U373MG-PMLIV cells were infected with SeV (40 HAU/ml) or EMCV (MOI of 0.5), or transfected with poly(I:C) (1 µg/ml). HeLa cells transduced with PMLIV-expressing or with noncoding parental (EV) lentiviral vectors were infected with HTLV-1 (HTLV, 600 ng/ml of p19-equivalent), influenza virus (Flu, 40 HAU/ml) or vaccinia virus (Vac, MOI of 2). After 8 h, mRNAs were extracted and IFN-βtranscripts quantified by RT-qPCR. (C) SUMOylation of PMLIV was required for IFN-β induction. U373MG-EV, U373MG-PMLIV, U373MG-PMLIV-3KR, U373MG-PMLIII and U373MG-PMLIII-3KR cells were infected with VSV for 12 h at an MOI of 0.2. Total RNA was extracted and mRNAs encoding IFN-β and GAPDH were quantified by RT-qPCR. Mean values and standard deviations of two independent experiments are shown.

To determine whether PMLIV affects the expression of IFNs and/or pro-inflammatory cytokines, we analyzed IFN-α, IFN-β, TNF-α and IL8 mRNAs by RT-qPCR in extracts from U373MG-EV and U373MG-PMLIV cells infected with VSV at an MOI of 0.2 for various lengths of time. PMLIV did not significantly alter the mRNA expression of IFN-α, TNF-α or IL8 following VSV infection (Figure 5A). Thus, PMLIV had no effect on the induction of TNF-α and IL-8 mRNAs that are regulated by NF-κB, or on the induction of IFN-α mRNA that is regulated by IRF7 [33]. In contrast, as early as 8 h post-infection, IFN-β mRNA expression that is known to occur through activated IRF3, was enhanced by PMLIV leading to as high as a 2-log increase 12 h post-infection. Like PMLIV, PMLIVa increased IFN-β mRNA synthesis upon VSV infection (data not shown). Thus, both PMLIV and its variant PMLIVa sharing the same C-terminal region unique to this isoform, boosted IFN-β induction upon VSV infection. Next, we asked whether PMLIV-dependent boost of IFN-β transcription was specific to VSV infection or whether it was a broader mechanism triggered by pattern recognition receptor (PRR) activation. To do this, U373MG-EV or U373MG-PMLIV cells were either infected with SeV (40HAU/ml) or transfected with poly(I:C) (1 µg/ml). After 8 h, mRNAs were extracted and IFN-βtranscripts quantified by RT-qPCR. As shown in Figure 5B, we found that PMLIV also enhanced IFN-β mRNA synthesis in U373MG cells infected with SeV, EMCV or transfected with poly(I:C) (Figure 5B). Also, HeLa cells transduced with PMLIV-expressing or with noncoding parental (EV) lentiviral vector and infected with HTLV-1, influenza or vaccinia virus revealed that PMLIV positively regulated IFN-β mRNA production (Figure 5B). This demonstrates that the ability of PMLIV to potentiate IFN-β synthesis is not a specific feature of VSV infection but a more general mechanism following RNA or DNA detection by PRRs.

### SUMOylation of PMLIV is required for both its antiviral property and enhanced IFN-β induction

We investigated the role of SUMOylation in the antiviral property of PMLIV by using PMLIV-3KR mutant in which the three major SUMO-target lysines were substituted with arginines. Double immunofluorescence analysis of PML and viral proteins revealed that PMLIV inhibited viral protein synthesis, whereas PMLIV-3KR did not (Figure S2A, left panel). PML and its SUMOylated forms were indeed produced in cells stably expressing PMLIV but, as expected, only the unmodified form was detected in cells stably expressing the SUMO-deficient PML mutant, PMLIV-3KR (Figure S2A, right panel). Western blot analysis of extracts from infected U373MG-EV, U373MG-PMLIV and U373MG-PMLIV-3KR cells confirmed that VSV protein synthesis was inhibited by PMLIV but was not affected by PMLIV-3KR (Figure S2B, left panel). In addition, VSV growth was inhibited in cells expressing PMLIV, but not in cells expressing PMLIV-3KR (Figure S2B, right panel). To determine the role of PMLIV SUMOylation on IFN-β synthesis, we quantified by RT-qPCR IFN-β mRNA in extracts from U373MG-EV, U373MG-PMLIV and U373MG-PMLIV-3KR cells infected with VSV (Figure 5C). As PMLIII also inhibits VSV multiplication, we also quantified IFN-β mRNA in extracts from U373MG-PMLIII and U373MG-PMLIII-3KR cells. Again, we observed a nearly 2 log increase of IFN-β mRNA expression in VSV infected cells expressing PMLIV. Strikingly, the induction was completely lost when the PMLIV-3KR was expressed, demonstrating that SUMOylation of PMLIV is also required for the enhancement of IFN-β mRNA expression. In contrast, following VSV infection, PMLIII did not increase the mRNA synthesis of IFN-β, IFN-α, TNF-α or IL8 (Figure 5C and data not shown). Taken together, these results demonstrate that SUMO modification of PMLIV is required to confer resistance towards VSV infection and also to increase IFN-β synthesis.

### PMLIV is the only PML isoform able to boost IFN-β synthesis through IRF3 activation

**Figure 6:**
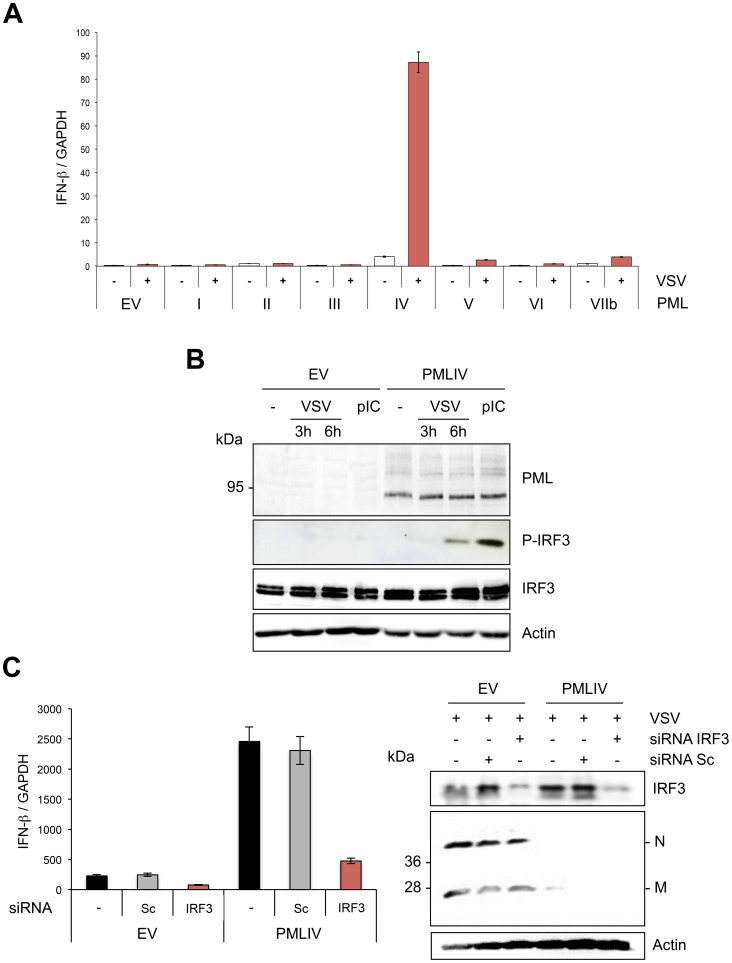
PMLIV is the only PML isoform able to boost IFN-β synthesis through IRF3 activation. (A) Quantification of IFN-β mRNA in infected cells expressing different PML isoforms. U373MG-EV cells or cells stably expressing PMLI, PMLII, PMLIII, PMLIV, PMLV, PMLVI or PMLVIIb were infected with VSV at an MOI of 0.2 for 12 h. Total RNA was extracted and mRNA encoding IFN-β and GAPDH quantified by RT-qPCR. Mean values and standard deviations of two independent experiments are shown. (B) IRF-3 phosphorylation in VSV-infected or poly(I:C)-transfected cells expressing PMLIV. U373MG-EV or U373MG-PMLIV cells were left untreated (-), infected with VSV at an MOI of 1 for 3 or 6 h or transfected with poly(I:C). Cell extracts were analyzed by Western blotting using antibodies directed against PML, P-IRF3, IRF3 or Actin. (C) Effect of IRF3 knockdown on VSV inhibition and IFN-β induction. U373MG-EV and U373MG-PMLIV cells, prepared in duplicate, were transfected with scramble (Sc) or IRF3-specific siRNA. Two days later, cells were infected with VSV at an MOI of 1 for 8 h. Total RNA was extracted in one series and mRNA encoding IFN-βand GAPDH was quantified by RT-qPCR (left panel). Means and standard deviations of three independent experiments are shown. The second series was used for the determination by Western blot of IRF3 and VSV protein expression (right panel).

To determine the effect of other PML isoforms on IFN-β synthesis, U373MG-EV cells and cells stably expressing each PML isoform (PMLI, PMLII, PMLIII, PMLIV, PMLV, PMLVI or PMLVIIb) were infected with VSV at an MOI of 0.2 and IFN-β mRNA was quantified in their extracts by RT-qPCR (Figure 6A). Among all PML isoforms, only PMLIV expression resulted to a dramatic increase of IFN-β mRNA synthesis following VSV infection, reaching a nearly 2 log rise. To rule out the possibility that this increase could be due to the stabilization of IFN-β encoding mRNA by PMLIV, we performed a transcriptional assay. To do this, we transfected control or PMLIV-expressing cells with a reporter plasmid containing the firefly luciferase gene under the control of the human IFN-β promoter and infected the cells with VSV. Using this reporter assay, we were able to confirm that PMLIV greatly enhances the transcription driven by the IFN-β promoter (data not shown), confirming that this increase is at the transcriptional level.

Among members of the IFN regulatory factor family, IRF3 plays an essential role in virus-induced *IFN-β* gene expression [34]. Interestingly, IFN produced by PMLIV-expressing cells upon VSV infection was due to activated IRF3, since a higher amount of phosphorylated IRF3 (P-IRF3) was detected 6 h post-infection of cells expressing PMLIV compared to control cells (Figure 6B). Interestingly, PMLIV also enhanced IRF3 phosphorylation in cells transfected with poly(I:C) (Figure 6B). In contrast, the level of IRF3 was not altered following VSV infection or poly(I:C) transfection. To further determine the role of IRF3 in anti-VSV activity and enhanced IFN synthesis, U373MG-EV and U373MG-PMLIV cells depleted for IRF3 were infected at an MOI of 1 for 8 h and their extracts were analyzed by RT-qPCR for IFN-β mRNA and by Western blot for IRF3 and viral protein expression. As seen in Figure 6C, in PMLIV expressing cells infected for 8 h, depletion of IRF3 abrogated IFN-β mRNA synthesis (Figure 6C, left panel) without affecting the inhibition of VSV protein synthesis (Figure 6C, right panel) and viral production (data not shown). This demonstrates that the intrinsic anti-VSV activity of PMLIV is independent of IRF3.

Thus, taken together, our results demonstrate that PMLIV is the only PML isoform able to inhibit VSV at a high MOI independently of IRF3 and also to stimulate IFN-β synthesis *via* an increase of IRF3 activation.

### Intrinsic and innate immune properties of PMLIV

**Figure 7:**
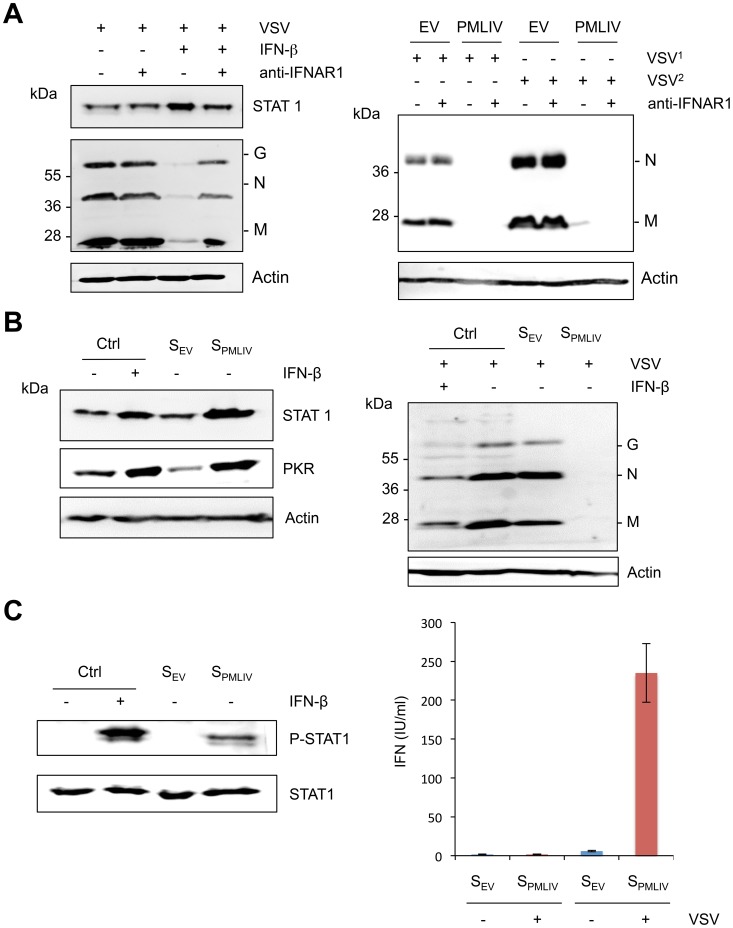
Intrinsic and innate immune properties of PMLIV. (A) U373MG cells were left untreated or treated for 24 h with 100 units/ml of IFN-β in the absence or the presence of 20 µg/ml of purified anti-IFNAR1 mAb before infection with VSV at an MOI of 1 for 8 h (left panel). U373MG and U373MG-PMLIV cells were infected with VSV at an MOI of 0.2 for 8 h (VSV^1^) or 12 h (VSV^2^) in the absence or the presence of 20 µg/ml of anti-IFNAR1 mAb (right panel). Cell extracts were analyzed by Western blotting and revealed by antibodies directed against VSV, STAT1 or Actin. (B) Activity of secreted IFN in infected U373MG-PMLIV cells. Culture supernatants (from U373MG-EV cells [S_EV_] and U373MG-PMLIV cells [S_PMLIV_]) were treated with acid buffer to inactivate virus, and the pH was then neutralized. One series of HeLa cells were left untreated as a control (Ctrl) or treated for 24 h with 100 units/ml of IFN-β, S_EV_ or S_PMLIV_, then their extracts were analyzed by Western blot for PKR and STAT1 (left panel). The second series of HeLa cells were infected with VSV at an MOI of 0.2 for 8 h and the cell extracts were analyzed by Western blotting using anti-VSV and anti-Actin antibodies (right panel). (C) Secreted IFN activates STAT1 phosphorylation (left panel). HeLa cells were left untreated (Ctrl) or treated for 30 min with 1000 units/ml of IFN-β, S_EV_ or S_PMLIV_, then their extracts were analyzed by Western blot for P-STAT1 and STAT1. Quantification of produced type I IFN upon VSV infection (right panel). IFN was quantified using the reporter cell line HL116 that carries the luciferase gene under the control of the IFN-inducible 6–16 promoter [55]. HL116 cells were incubated for 8 h with a standard containing titrated human IFN-β, the supernatants from U373MG-EV [S_EV_] and U373MG-PMLIV [S_PMLIV_] cells infected with VSV at an MOI of 1 for 20 h or the supernatants from uninfected U373MG-EV and U373MG-PMLIV. Cells were then lysed and luciferase activity measured. IFN levels were expressed as equivalent of IFN-β concentration, in IU/ml. Means and standard deviations of two independent experiments are shown.

PMLIV conferred viral resistance in cells 8 h or 12 h post-infection (Figure 3). This resistance was correlated with an induction of IFN-β mRNA synthesis observed as early as 8 h and increased as high as 2 log at 12 h post-infection (Figure 5). To determine whether or not the observed antiviral effect of PML at these times of infection was a secondary response to IFN synthesis, we tested the capacity of PMLIV to inhibit VSV in cells treated with an anti-IFNAR1 mAb targeting the extracellular domain of the IFNAR1 chain of the human IFN-α/β receptor. The anti-IFNAR1 mAb inhibits the binding and biological activity of type I IFN [35] as well as the IFN-β-induced STAT1 expression and anti-VSV activity (Figure 7A, left panel). In contrast, this antibody did not alter the inhibition of VSV protein synthesis by PMLIV in cells infected for either 8 h or 12 h (Figure 7A, right panel). Since STAT1 is the central transcription factor required for the biological responses of all types of IFN, we determined the effect of its downregulation on PMLIV-induced VSV resistance (Figure S2C). The capacity of PMLIV to inhibit VSV protein synthesis was still maintained in cells depleted for STAT1 (Figure S2C), further demonstrating the intrinsic anti-VSV effect of PMLIV at 12 h post-infection. Collectively, these results demonstrate that PMLIV exerts an early intrinsic anti-VSV activity that is independent of IFN.

To determine whether the IFN produced and secreted after a longer period of infection was active, culture supernatants from U373MG-EV cells (S_EV_) and U373MG-PMLIV cells (S_PMLIV_) infected with VSV for 20 h were tested, in comparison with IFN-β, for their capacities to induce ISG products and to inhibit viral protein synthesis. Therefore, HeLa cells treated with medium (Ctrl), IFN-β, S_EV_ or S_PMLIV_ supernatant for 24 h were uninfected (Figure 7B, left panel) or infected with VSV for 8 h (Figure 7B, right panel). As seen in Figure 7B (left panel), STAT1 and PKR expression was increased only in cells treated with IFN-β and S_PMLIV_ supernatant. In addition, VSV proteins were inhibited only in extracts from cells pretreated with IFN-β or S_PMLIV_ supernatant (Figure 7B, right panel). It should be noted that the capacity of S_PMLIV_ supernatant to induce STAT1 and PKR expression as well as to inhibit VSV was higher than that observed with 100 units/ml of IFN-β. In addition, S_PMLIV_ but not S_EV_ supernatant was able to induce STAT1 phosphorylation in HeLa cells (Figure 7C, left panel). Taken together, these results show that the IFN produced and secreted by U373MG-PMLIV cells 20 h post-VSV infection, activates STAT1, induces ISG products and protects HeLa cells from viral infection.

Next, we quantified the amount of type I IFN in supernatants from VSV-infected U373MG-EV and U373MG-PMLIV cells using the human HL116 cell line carrying the luciferase gene under control of the IFN-inducible 6–16 promoter. This experiment showed that PMLIV boosted the amount of type I IFN synthesized in infected cells by up to 200 to 300 international units/ml (IU/ml), depending on the experiment. A typical experiment is presented in Figure 7C (right panel). In addition, S_EV_ and S_PMLIV_ supernatants were also titrated on HeLa cells. The IFN titer of S_EV_ was below the detection limit (less than 2 IU/ml) and that of S_PMLIV_ was 250 IU/ml.

Taken together, these results demonstrate that PMLIV has a dual effect on viral infection: (i) an early intrinsic anti-VSV activity that was not eradicated by treatment with anti-IFNAR1 mAb, knockdown of STAT1 or IRF3 and (ii) an activation of innate immune signaling that occurs later and leads to the production and the secretion of type I IFN, which can protect other cells from viral infection.

### PMLIV interacts with Pin1 and recruits it within PML NBs

**Figure 8:**
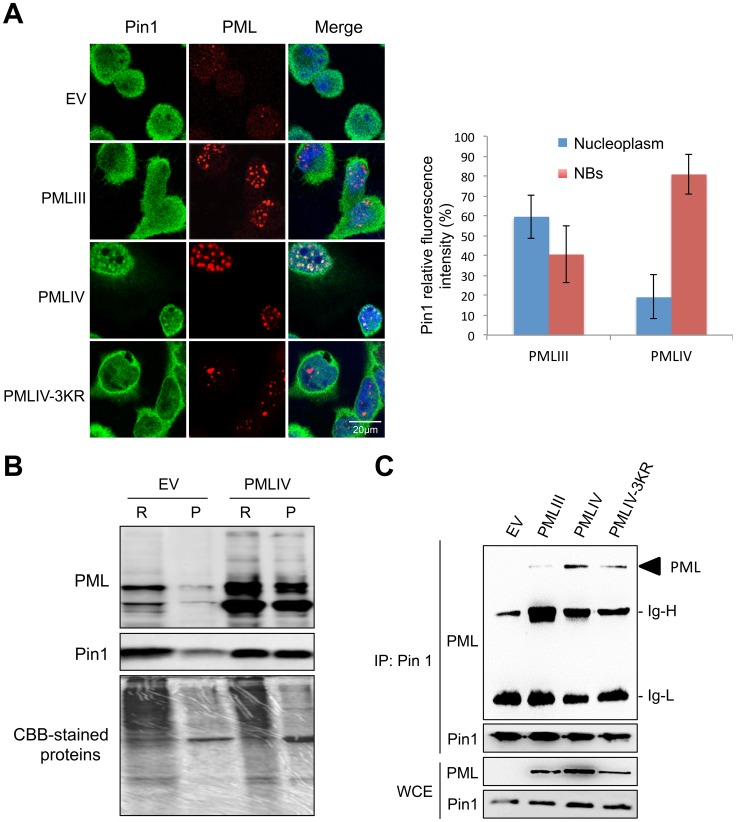
PMLIV interacts with Pin1 and recruits it within PMLNBs. (A) Double immunofluorescence analyses were performed using monoclonal anti-PML (red) and rabbit anti-Pin1 (green) antibodies in U373MG-EV cells or cells stably expressing PMLIII, PMLIV or PMLIV-3KR (left panel). Images obtained in PMLIII- and PMLIV-expressing cells were quantified using Image-J software. The resulting relative values (mean ± s.e.m.) corresponding to Pin1 localization are shown in histograms (*n* = 30) (right panel). (B) PMLIV induces the recruitment of Pin1 to the nuclear matrix. RIPA soluble (R) and RIPA insoluble fractions (P) from U373MG-EV and U373MG-PMLIV cells were analyzed by Western blotting using anti-PML and anti-Pin1 antibodies. (C) PMLIV and PMLIV-3KR interacted with Pin1. Whole-cell lysates from U373MG-EV cells and cells expressing PMLIV, PMLIV-3KR or PMLIII were immunoprecipitated with anti-Pin1 antibodies. The immunopellets were separated by SDS-PAGE and immunoblotting was performed with anti-PML and anti-Pin1 antibodies. Ten percent input is shown (WCE).

Pin1 is known to interact with and to promote phosphorylated IRF3 degradation [30]. Since PMLIV increased IRF3 activation upon VSV infection, we asked whether PMLIV can specifically recruit endogenous Pin1 within PML NBs. Double immunofluorescence studies were performed on endogenous Pin1 and PML in cells expressing PMLIII, PMLIV or PMLIV-3KR. In both uninfected and infected cells endogenous Pin1 was found both in the cytoplasm and the nucleus (Figure 8A and data not shown). Importantly, Pin1 was found colocalizing with PMLIV within the NBs in uninfected or VSV-infected cells (Figure 8A and data not shown). Indeed, Pin1 was found diffuse in the nucleus of EV, PMLIII and PMLIV3KR cells, whereas in PMLIV-expressing cells, it formed speckles colocalizing with PML NBs. Interestingly, such colocalization was not observed in cells expressing PMLIII or PMLIV-3KR (Figure 8A, left panel). Fluorescence intensities were quantified using Image-J software and revealed that the portion of Pin1 associated to PML NBs highly increased in PMLIV-expressing cells (Figure 8A, right panel). Thus, PMLIV induced a relocalization of Pin1 from the nucleoplasm to the NBs.

**Figure 9:**
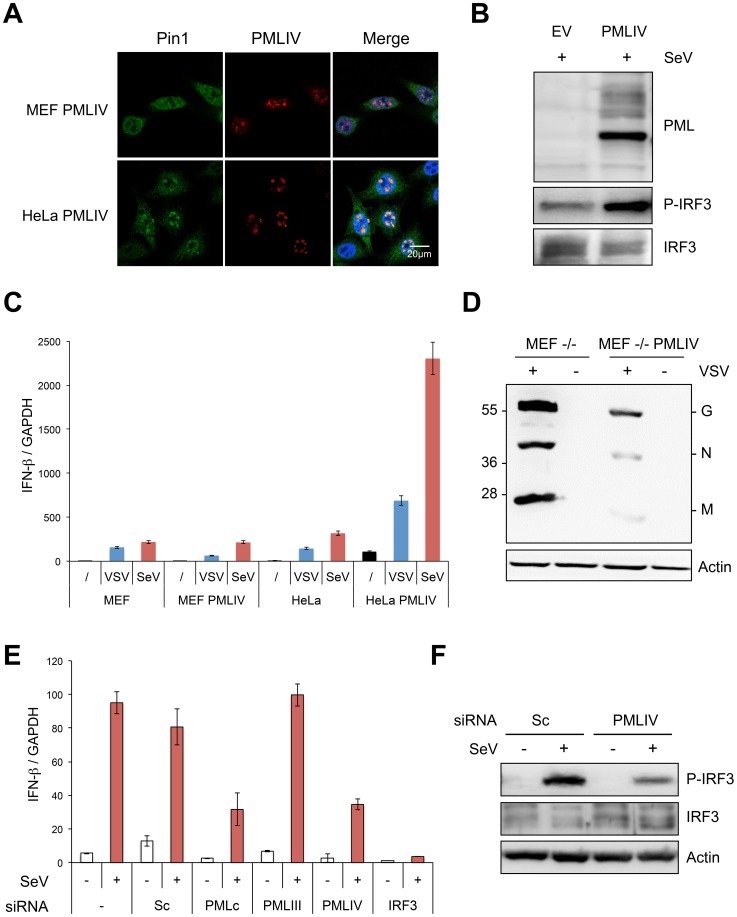
PMLIV is responsible for Pin1 recruitment in PML NBs, enhanced P-IRF3 and IFN-β synthesis upon viral infection. (A) PMLIV recruits Pin1 within the NBs in HeLa cells but not in MEFs. MEFs or HeLa cells were transduced with HIV-derived lentiviral vectors expressing PMLIV. Two days post-transduction, double immunofluorescence analyses were performed using monoclonal anti-PML (red) and rabbit anti-Pin1 (green) antibodies. (B) HeLa cells transduced with HIV-derived lentiviral vectors expressing EV or PMLIV were infected with SeV (40 HAU/ml) for 8 h and their extracts were analyzed by Western blot for PML, P-IRF3 and IRF3 expression. (C) Wild-type MEF and HeLa cells transduced with PMLIV-expressing or with the noncoding parental lentiviral vector (EV) were infected with VSV (MOI of 0.2) for 12 h or with SeV (40 HAU/ml) for 8 h. After RNA extraction, GAPDH and IFN-β transcripts were quantified by RT-qPCR. Mean values and standard deviations of two independent experiments are shown. (D) MEF PML-/- cells were transduced with pTRIP-PMLIV and infected with VSV at a MOI of 1 for 8 h. Cell extracts were analyzed by Western blot and revealed using anti-VSV and anti-actin antibodies. (E) U373MG cells were transfected with scramble siRNA (Sc), or with a siRNA targeting IRF3, PMLIII, PMLIV or common to all PML isoforms (PMLc), before infection with SeV (40 HAU/ml) for 12 h. After RNA extraction, GAPDH and IFN-β transcripts were quantified by RT-qPCR. Mean values and standard deviations of two independent experiments are shown. (F) Extracts of U373MG cells transfected with siRNA (Sc), or with siRNA specific to PMLIV before infection with SeV, were analyzed by Western blot for P-IRF3, IRF3 and Actin expression.

The recruitment of Pin1 to PML NBs by PMLIV was further demonstrated by Western blot analysis of the RIPA soluble and insoluble fractions (Figure 8B). In EV cells, most of the Pin1 was found in the RIPA-soluble fraction that included both the cytoplasm and the nucleoplasm, whereas a small fraction was associated to the nuclear matrix (RIPA-insoluble fraction). As observed by immunofluorescence, the expression of PMLIV resulted in a shift of Pin1 to the nuclear matrix, resulting in an enrichment of Pin1 in the RIPA insoluble fraction (Figure 8B). Co-immunoprecipitation assays revealed that PMLIV and PMLIV-3KR interacted with endogenous Pin1 whereas a very slight interaction was detected with PMLIII (Figure 8C). The recruitment of Pin1 within PML NBs was observed in various human cell lines including HeLa cells transduced with a lentiviral vector expressing PMLIV (Figure 9A, and data not shown). The recruitment of Pin1 within PML NBs by PMLIV in HeLa cells was also associated with a positive regulation of both IRF3 activation (Figure 9B and data not shown) and IFN-β synthesis upon VSV or SeV infection (Figure 9C). Thus, PMLIV expression in different infected human cells resulted in enhanced IRF3 phosphorylation and IFN-β mRNA production (Figures 5, 6 and 9). At the opposite, the expression of PMLIV in MEF cells neither induced the recruitment of endogenous mouse Pin1 within NBs (Figure 9A) nor enhanced IRF3 activation or IFN-β synthesis upon viral infection (Figure 9C and data not shown). Our results suggest that the recruitment of Pin1 by PMLIV within PML NBs is required for PMLIV-induced enhancement of IFN-β synthesis.

Next, we asked whether the intrinsic anti-VSV activity of PMLIV was observed in PML-/-MEF cells. To do this, PML-/- MEFs were transduced with EV- or PMLIV-encoding lentivector before infection with VSV. As seen in Figure 9D, PMLIV was still able to inhibit VSV protein synthesis when expressed in PML-/- cells, thus revealing that the intrinsic anti-VSV activity of PMLIV did not require the expression of endogenous PML.

To further confirm the specific positive regulation of PMLIV on IFN-β transcription, U373MG cells were infected with SeV 48 h after transfection with scramble siRNA (Sc), siRNA targeting IRF3, siRNA common to all PML isoforms (siRNA PMLc), or with the siRNA specific to PMLIII or to PMLIV that we have previously validated [23]. As expected, the siRNA IRF3 completely abolished IFN-β mRNA synthesis upon SeV infection (Figure 9E). SeV-induced IFN-β expression was not altered by PMLIII depletion but was highly reduced following the depletion of all PML isoforms or the specific depletion of PMLIV (Figure 9E). Furthermore, specific suppression of PMLIV expression reduced SeV-induced IRF3 activation (Figure 9F). This demonstrates that endogenous PMLIV is required for the efficient synthesis of IFN-β transcription upon viral infection and validates our data obtained in cells overexpressing PMLIV.

Taken together, these results show that PMLIV and PMLIV-3KR interacted with Pin1 but only PMLIV was able to recruit it within PML NBs where both proteins colocalized. Therefore, the interaction of endogenous Pin1 with SUMOylated PMLIV and its recruitment in PML NBs could alter Pin1-induced downregulation of activated IRF3 thus resulting in a higher amount of phosphorylated IRF3 during viral infection. In addition, the intrinsic anti-VSV activity of PMLIV is independent of the expression of other PML isoforms.

## Discussion

**Figure 10:**
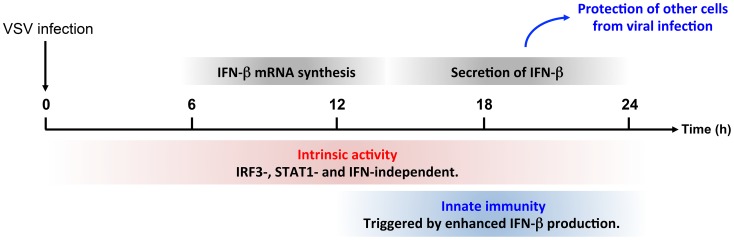
Intrinsic and innate immune properties of PMLIV during viral infection. The anti-VSV intrinsic activity of PMLIV is independent of IRF3, STAT1 and IFN and can be observed during the whole duration of the experiments. In parallel, PMLIV enhances IFN-β mRNA synthesis around 12 h post-infection. This produced IFN is secreted between 18 and 24 h and then acts on other cells by activating STAT1, increasing the expression ISG products thus protecting them from uncoming infection.

Many reports implicate PML and PML NBs in antiviral responses targeting diverse cytoplasmic replicating RNA viruses through different mechanisms [13], [17], [19], [21], [23]–[25], [36]. An antiviral effect of PML against *rhabdoviridae* has been observed *in vivo*, as PML deficiency renders mice more susceptible to VSV infection [14]. In this report, we show that cells derived from these mice or human cells depleted of PML produced a higher level of viral proteins. Among the various PML isoforms tested, only stable expression of PMLIII and PMLIV conferred resistance to VSV. This inhibitory effect did not alter VSV entry, but was observed at the level of viral mRNA and protein synthesis, resulting in a reduction of VSV yields and in cell lysis protection. The protective property of PMLIV was found to be higher than that of PMLIII, as PMLIV was able to inhibit virus growth up to an MOI of 2, resulting in a 500-fold reduction of VSV yields. Whereas PMLIII confers viral resistance in an IFN-independent way, PMLIV displays two antiviral activities during VSV infection: an early IFN-independent activity targeting VSV replication followed by the activation of innate immunity pathways, leading to an enhanced type I IFN synthesis, which protects yet uninfected cells from viral infection (Figure 10). Interestingly, PMLIV-3KR failed to confer resistance to VSV and also to induce IFN-β synthesis, demonstrating that SUMOylation of PMLIV is required for both intrinsic antiviral activity and innate immune property.

The induction of IFN-β expression is the key event in the initiation of the innate antiviral response. Central to this process is the activation of IRF3 *via* its phosphorylation [34]. Here we demonstrate for the first time that one particular isoform of PML (PMLIV) is implicated in innate immunity, triggering a dramatic increase of IFN-β synthesis *via* IRF3 phosphorylation upon VSV infection. Depletion of IRF3 further demonstrates the dual activity of PMLIV, as it abrogated PMLIV-induced IFN synthesis but not PMLIV-induced inhibition of viral replication.

Pin1 was shown to interact with phosphorylated IRF3 and to promote its ubiquitin-mediated proteasomal degradation [30]. We report here that endogenous Pin1 interacts with PMLIV and that both proteins colocalize in PML NBs. This results in sustained IRF3 activation and higher IFN-β induction during VSV infection. Thus, the recruitment of endogenous Pin1 in PML NBs might antagonize Pin1-induced P-IRF3 degradation [30] and could be a novel mechanism for inhibiting Pin1 function. Consistently, host antiviral responses are boosted in the presence of PMLIV. Collectively, our report characterizes PMLIV as a positive regulator of antiviral innate immune responses, which maintains stability of IRF3 phosphorylation through the interaction of endogenous Pin1 with SUMOylated PMLIV and its recruitment in PML NBs.

Thus, our results suggest that, whereas the direct antiviral activity of PMLIV is specifically targeting VSV, the positive regulation of innate immunity should also be observed using other stimuli than VSV infection since PMLIV also recruited Pin1 within the NBs in uninfected cells. Indeed, we observed that IRF3 activation and IFN-β production were also drastically enhanced in PMLIV-overexpressing cells following transfection with poly(I:C) or infection with viruses from different families such as SeV, EMCV, HTLV-1, influenza virus or vaccinia virus. These observations confirm that PMLIV has a specific anti-VSV activity and a much wider positive effect on innate immunity pathways. Remarkably, specific depletion of endogenous PMLIV in human U373MG or HeLa cells reduced VSV- as well as SeV-induced activation of IRF-3 and consequent production of IFN-β (this paper and data not shown).

This is the first demonstration of the implication of PML in the enhancement of IFN-β production upon viral infection. In contrast, the intrinsic antiviral activity of PML has been documented to act on viruses from different families [17], [21]. In the case of EMCV, PMLIV is the only PML isoform that confers viral resistance, by interacting with the viral polymerase 3D, and sequestering it within PML NBs where both proteins colocalize [23]. Interestingly, PMLIV is also able to sequester the ORF23 capsid protein within PML NBs, leading to the inhibition of varicella-zoster virus (VZV) an alphaherpesvirus [37]. PML has also been shown to interfere with retrovirus replication, since PMLIII interacts with Tas, the transcriptional transactivator of human foamy virus, resulting in viral restriction [24]. The mechanism by which the nuclear PMLIV directly inhibits VSV, whose replication takes place entirely in the cytoplasm, remains to be elucidated. We demonstrated that the intrinsic anti-VSV property of PMLIV did not require the expression of other PML isoforms and was independent of IFN since it was maintained in cells treated with antibodies against type I IFN receptors, depleted for IRF3 or depleted for STAT1. However, further investigations are needed to demonstrate how PMLIV and PMLIII exert their intrinsic anti-VSV activity by interacting with a viral or a cellular protein required for VSV replication. Understanding the intrinsic antiviral activity of PML may introduce new ways for targeted antiviral therapy that would bypass the need for IFN treatment.

Thus, PML confers viral resistance in two ways (Figure 10). It can exert an intrinsic anti-VSV activity independent of IRF3, STAT1 and IFN. In addition, we show here that PML enhanced both IRF3 activation and IFN-β synthesis upon viral infection. This produced IFN-β is secreted and protects yet uninfected cells from oncoming infection.

Our results demonstrate that PML, an ISG product with a broad intrinsic antiviral activity, is also able to trigger IFN-β synthesis upon viral infection. There are an increasing number of ISG products that are also implicated in innate immunity processes. PKR for example, long known to mediate the antiviral activities of IFNs, also plays an important role in the induction of type I IFN, particularly IFN-β during measles virus infection [38] or double-stranded RNA treatment [39]. Indeed, activation of PKR increases IRF3 activation, and knockdown of PKR reduces both activated IRF3 level and IFN-β induction [39]. The role of the endoribonuclease RNase L in the innate antiviral immune response has been demonstrated *in vivo*. Indeed, injection of 2′5′-linked oligoadenylates leads to IFN-β synthesis in wild-type mice but not in RNase L deficient mice. In addition, EMCV- or Sendai virus-induced IFN is highly reduced in mice lacking RNase L [40].

Interestingly, many members of the TRIM protein family, which PML belongs to, (i) are products of ISGs [41], (ii) display a direct antiviral activity [42] and/or have been identified as important players of innate immunity [43]. TRIM5α for instance is induced by type I IFN [44], [45], inhibits retroviral infections [46] and is also able to promote innate immune signaling [47]. TRIM25 protein plays a key role in innate immunity, since it is essential for RIG-I-mediated antiviral activity [48]. Strikingly, TRIM21 is also able to enhance IRF3-mediated antiviral response. Indeed, TRIM21 was shown to interact directly with IRF3 upon viral infection and to interfere with its interaction with Pin1 [49]. Recent studies performed on the entire TRIM protein family allowed the identification of several other members of this family as key components of inflammation and innate immunity signaling pathways [50], [51]. Our results show that TRIM19/PML, another member of the TRIM protein family, acts, through Pin1 recruitment in PML NBs, as a positive regulator of IRF3 phosphorylation, enhancing the strength and duration of IFN-β-induced antiviral response. Thus, PML can therefore be added to the list of TRIM proteins implicated in both intrinsic and innate immunity. Initially considered as two independent arms of the immune system, our results further suggest a closer crosstalk between intrinsic and innate immunity.

## Materials and Methods

### Materials

Human recombinant IFN-β was purchased from Biogen Inc. Rabbit polyclonal (sc-5621) and mouse anti-PML (sc-966), rabbit anti-IRF3 (sc-9082), rabbit anti-STAT1 (sc-345), rabbit anti-phospho-STAT1 (Tyr701, sc-7988) and rabbit anti-PKR antibodies (sc-707) were obtained from Santa-Cruz Biotechnology. The rabbit anti-phospho-IRF3 (Ser 396) and rabbit anti-Pin1 antibodies were obtained from Cell Signaling, and HRP-conjugate monoclonal anti-Actin antibody from Sigma. The 64G12 monoclonal antibody against human IFN-α/β receptor (anti-IFNAR1 mAb) was a gift from P Eid (INSERM UMR1014) [35]. The rabbit anti-VSV polyclonal antibodies (home-made) were obtained by repeated injection of purified virus. Reactivities against the viral proteins N, M or G were different depending on the batch used in western-blot experiments.

### Cell cultures

Human glioblastoma astrocytoma U373MG, epithelial HeLa and fibrosarcoma HL116 cells as well as mouse embryonic fibroblasts (MEFs) from wild-type (WT) or knockout PML (PML-/-) mice [52], were grown at 37°C in DMEM supplemented with 10% FCS. U373MG cells transfected with empty vector or stably expressing individual PML isoforms (PMLI to VIIb), PMLIII-3KR or PMLIV-3KR were kept in medium supplemented with 0.5 mg/ml of neomycin. HL116 cells were grown in medium supplemented with HAT (Hypoxanthine: 20 µg/ml, Aminopterin: 0.2 µg/ml, Thymidine: 20 µg/ml).

### Stable expression of PML isoforms

The accession number (GenBank) for PML isoforms are AF230401 (PMLI), AF230403 (PMLII), S50913 (PMLIII), AF230406 (PMLIV), AF230411 (PMLIVa), AF230402 (PMLV), AF230405 (PMLVI), AF230408 (PMLVIIb). In PMLIII-3KR and PMLIV-3KR mutants, the three SUMO-target lysines (at positions 65, 160, and 490) were replaced with arginines. Stable U373MG cells expressing each of the PML isoforms (PMLI to VIIb), PMLIII-3KR or PMLIV-3KR were obtained *via* transfection with constructs corresponding to each cloned in pcDNA3.1 and subsequent neomycin selection at a final concentration of 0.5 mg/ml [25]. Control U373MG cells were generated in the same way using the empty vector (U373MG-EV). For expressing PMLIV in MEFs wild-type, MEFs PML-/- and HeLa cells, we constructed an HIV-derived lentiviral vector (pTRIP-PMLIV), which was used to transduce the cells. The pTRIP plasmid was provided by P Charneau (Institut Pasteur, Paris, France) [53].

### Viral stocks

VSV (Mudd-Summer strain, Indiana serotype) was grown in BSR cells. BSR cells were infected at an MOI of 0.1. After 24 h, supernatants were collected and cellular debris removed by low-speed centrifugation. Virus titers (10^9 ^PFU/ml) were determined by standard plaque assay onto BSR cells. EMCV was produced as described [20] and has a titer value of 2.10^8 ^PFU/ml. Vaccinia virus has a titer of 2.10^9 ^PFU/ml. Sendai virus was kindly provided by E Meurs (Institut Pasteur, Paris, France). It was used at 40 HAU/ml to activate RIG-I, as described [54]. HTLV-1, and influenza A virus (strain A/PR 8/34) were provided by JP Herbeuval (CNRS UMR 8601). HTLV-1 and influenza virus were used at 600 ng/ml of p19-equivalent and at 40 HAU/ml, respectively. MLV-derived vectors encoding GFP pseudotyped with VSV-G, provided by FL Cosset (ENS Lyon), had a titer value of 10^7^ IU/ml and were generated as described [31].

### siRNA transfection

Cells were seeded in six-well plates and transfected with siRNA using Lipofectamine RNAiMax transfection reagents (Invitrogen). The mRNA sequence targeted by the siRNA PMLc (common to all PML isoforms) is 5′-AUGGCUUCGACGAGUUCAATT-3′, by siRNA PMLIII is 5′-AGUGCAUGGAGCCCAUGGATT-3′ and by siRNA PMLIV is 5′UGAAAGUGGGUUCUCCUGGTT-3′. The siRNA scramble sequence is the following: 5′-GCAUGAACCGGAGGCCCAUUU-3. STAT1 or IRF3 expression was silenced using ON-TARGETplus SMARTpool siRNAs purchased from Thermo Scientific.

### Northern blot analysis

Cells were infected with VSV at an MOI of 3 in the absence or the presence of cycloheximide (100 µg/ml). After adsorption for 1 h, cells were washed and fresh medium with or without cycloheximide (100 µg/ml) was added. After 4 h, total RNA was isolated from cells with the RNA NOW Kit (Ozyme). Total RNA was separated on 1.5% agarose gel under denaturing conditions and blotted onto Nylon membranes (Roche Molecular Biochemicals). Hybridizations were performed with digoxigenin (DIG)-labeled oligonucleotides recognizing the VSV-N gene sequence and by incubation with anti-DIG antibody conjugated to alkaline phosphatase followed by CDP Star.

### Real-time PCR

Total RNA was extracted using RNeasy Mini Kit (Qiagen) and cDNAs were prepared using Oligo(dT) primer and SuperScript II Reverse Transcriptase (Invitrogen). Real-time PCR reactions were performed in duplicates using Platinum SYBR Green qPCR SuperMix-UDG (Invitrogen) following manufacturer's instructions. GAPDH, IFN-α1, IFN-β, TNF-α, IL8 and VSV-L encoding cDNAs were amplified on a Mastercycler ep realplex (Eppendorf) with a denaturation step of 5 min at 95°C followed by thirty-five cycles of 10 s at 95°C, 10 s at 60°C and 20 s at 72°C. Threshold cycle (Ct) values were converted to 2^−Ct^ in order to be proportional to the amount of transcripts in the samples. To compare samples, 2^−ΔCt^ were calculated by normalizing the data by the expression of GAPDH: 2^−ΔCt^ = 2^−Ct^(sample)/2^−Ct^(GAPDH). Primers used for quantification of transcripts by real time quantitative PCR are the following: VSV L (Forward: TGATACAGTACAATTATTTTGGGAC and Reverse: GAGACTTTCTGTTACGGGATCTGG), GAPDH (Forward: ACTTCAACAGCGACACCCACT and Reverse: GTGGTCCAGGGGTCTTACTCC), IFN-α1 (Forward: CCAGTTCCAGAAGGCTCCAG and Reverse: TCCTCCTGCATCACACAGGC), IFN-β (Forward: TGCATTACCTGAAGGCCAAGG and Reverse: AGCAATTGTCCAGTCCCAGTG), TNF-α (Forward: GGCGTGGAGCTGAGAGATAAC, Reverse: GGTGTGGGTGAGGAGCACAT) and IL-8 (Forward: AAGGGCCAAGAGAATATCCGAA and Reverse: ACTAGGGTTGCCAGATTTAACA).

### Immunofluorescence analysis

Immunofluorescence analyses were performed as described [23]. PML was detected with mouse anti-PML antibody and the corresponding anti-IgG antibody conjugated to Alexa 594. The VSV antigens or Pin1 protein were detected with rabbit anti-VSV or anti-Pin1 antibodies followed by Alexa 488. The cells were mounted onto glass slides by using Immu-Mount (Shandon) containing 4,6-diamidino-2-phenylindole (DAPI) to stain nuclei. Confocal laser microscopy was performed on a Zeiss LSM 510 microscope.

Quantitative analysis of immunofluorescence data was carried out by histogram analysis of the fluorescence intensity at each pixel across the images using Image J software. The results of the analysis of 20 images acquired in each experimental condition were then combined to allow quantitative estimates of changes in Pin1 localization.

### Cell fractionation and western blot analysis

Cells were washed and re-suspended in PBS, lysed in hot Laemmli sample buffer and boiled for 5 min. For cell fractionation, cells were dissociated and washed twice in PBS. The cytoplasmic and nucleoplasm fractions were extracted by incubating the cell pellet in 50 µl of RIPA buffer for 20 min on ice followed by centrifugation at 15000 g for 15 min to separate the RIPA soluble fraction (R) from the pellet (P). This RIPA insoluble fraction (P) was suspended in 50 µl of PBS. Fifty µl of 2X Laemmli buffer were added to each fraction, and the samples were boiled for 5 min before Western blot analysis. Protein extracts were analyzed on a 10% SDS-PAGE gel, and transferred onto a nitrocellulose membrane. The proteins were blocked on the membranes with 5% skimmed milk in PBS for 2 h and incubated overnight at 4°C with rabbit polyclonal anti-PML (clone H-238), anti-VSV, anti-IRF3, anti-phospho-IRF3 or anti-Actin antibodies. The blots were then washed extensively in PBS-Tween and incubated for 1 h with the appropriate peroxidase-coupled secondary antibodies (Cell Signaling Technology). All of the blots were revealed by chemiluminescence (ECL, Bio-Rad).

### Immunoprecipitation assays

Cells (10^7^) were incubated for 30 min at 4°C in 0. 5 ml of buffer containing 20 mM Tris-HCl pH 7.4, 1 M NaCl, 5 mM MgCl_2_, 1% triton, and 1 mM phenylmethylsulfonyl fluoride (PMSF). After cell lysis, 1.25 ml of immuno-precipitation buffer (IB) (20 mM Tris-HCl pH 7.4, 150 mM NaCl, 0.5% DOC, 1% Triton X-100, 0.1% SDS and 1 mM EDTA) were added. Rabbit anti-Pin1 antibodies were added and the samples incubated overnight at 4°C. Protein G beads (Sigma) in IB were then added and the samples mixed 2 h at room temperature. The beads were collected, washed four times with IB buffer and bound proteins were subjected to Western blotting.

### IFN quantification

To inactivate the virus, culture supernatants from infected cells were brought to pH 2 for 24 h and neutralized before titration. IFN secretion was quantified using the reporter cell line HL116 that carries the luciferase gene under the control of the IFN-inducible 6–16 promoter [55]. HL116 cells (2×10^4^) were plated in 96-well plate and incubated for 8 h with the desired culture supernatants or a standard of human IFN-β reference (Gb-23-902-531). Cells were then lysed (Luciferase Cell Culture Lysis Reagent, Promega) and luciferase activity measured using Perkin Elmer Wallac 1420. IFN titers were also quantified on HeLa cells challenged with VSV. IFN titers, defined as the amount of IFN leading to 50% inhibition of the cytopathic effect, were expressed in international unit/ml relative to the human IFN-β reference (Gb-23-902-531) of the NIH.

### Accession numbers

PMLI (AF230401), PMLII (AF230403), PMLIII (S50913), PMLIV (AF230406), PMLIVa (AF230411), PMLV (AF230402), PMLVI (AF230405), PMLVIIb (AF230408), STAT1

## Supporting Information

Figure S1 VSV protein synthesis in infected cells expressing each PML isoform. (A) Expression profile of PML in U373MG clones stably expressing PMLI, PMLII, PMLIII, PMLIV, PMLV or PMLVIIb, as revealed by Western-blotting using anti-PML and anti-Actin antibodies. (B) U373MG-EV cells or cells stably expressing each PML isoform were infected with VSV at different MOIs for 8 h. Extracts from these cells, non infected (-) or infected, were analyzed by Western blotting using anti-VSV and anti-Actin antibodies.
(TIF)

Figure S2 PMLIV SUMOylation is required for antiviral property. U373MG-EV, U373MG-PMLIV or U373MG-PMLIV-3KR cells were infected with VSV at an MOI of 1 for 8 h. (A/B) Double immunofluorescence staining was performed using monoclonal anti-PML (red) and rabbit anti-VSV (green) antibodies (A, left panel). Cell extracts were analyzed by Western blotting and revealed by antibodies directed against PML (A, right panel), VSV (B, left panel) or Actin. Supernatants from infected U373MG-EV, U373MG-PMLIV or U373MG-PMLIV-3KR cells were used for the determination of the virus yields (B, right panel). Means and standard deviations of two independent experiments are shown. (C) STAT1 depletion does not alter the intrinsic antiviral effect of PMLIV. U373MG-EV and U373MG-PMLIV cells were transfected with scramble (Sc) siRNA or STAT1-specific siRNA. Two days later, cells were infected with VSV at an MOI of 0.2 for 12 h. Cell extracts were used for the determination by Western blot of STAT1 and VSV protein expression.
(TIF)
