# Supplementary figures and images for: Implication of PMLIV in Both Intrinsic and Innate Immunity
Source: PLoS Pathog. 2014 Feb 27;10(2):e1003975. doi: 10.1371/journal.ppat.1003975 (PMC3937294; doi:10.1371/journal.ppat.1003975)

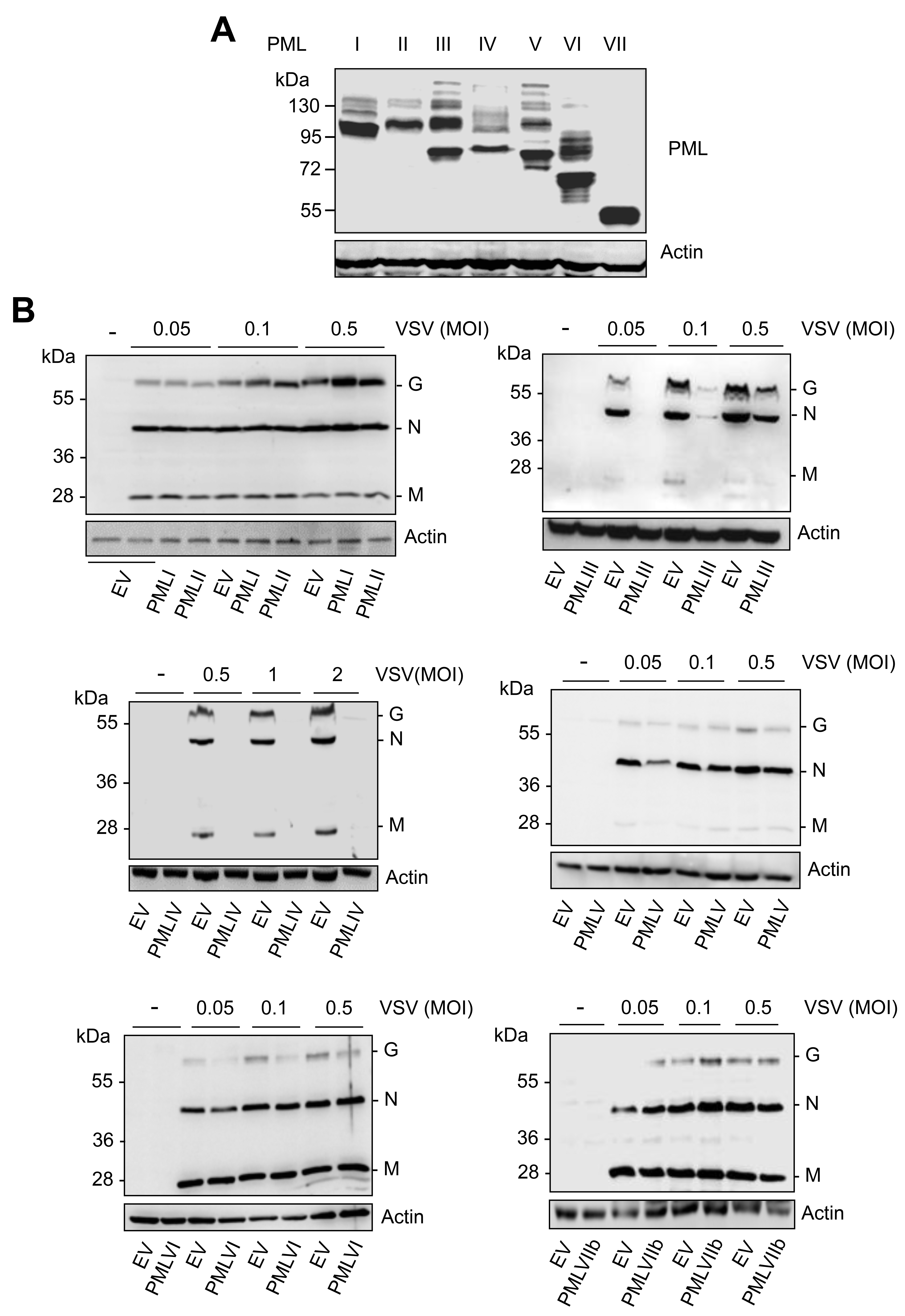

Supplement: Figure S1 — VSV protein synthesis in infected cells expressing each PML isoform. (A) Expression profile of PML in U373MG clones stably expressing PMLI, PMLII, PMLIII, PMLIV, PMLV or PMLVIIb, as revealed by Western-blotting using anti-PML and anti-Actin antibodies. (B) U373MG-EV cells or cells stably expressing each PML isoform were infected with VSV at different MOIs for 8 h. Extracts from these cells, non infected (-) or infected, were analyzed by Western blotting using anti-VSV and anti-Actin antibodies. (TIF) [file ppat.1003975.s001.tif]

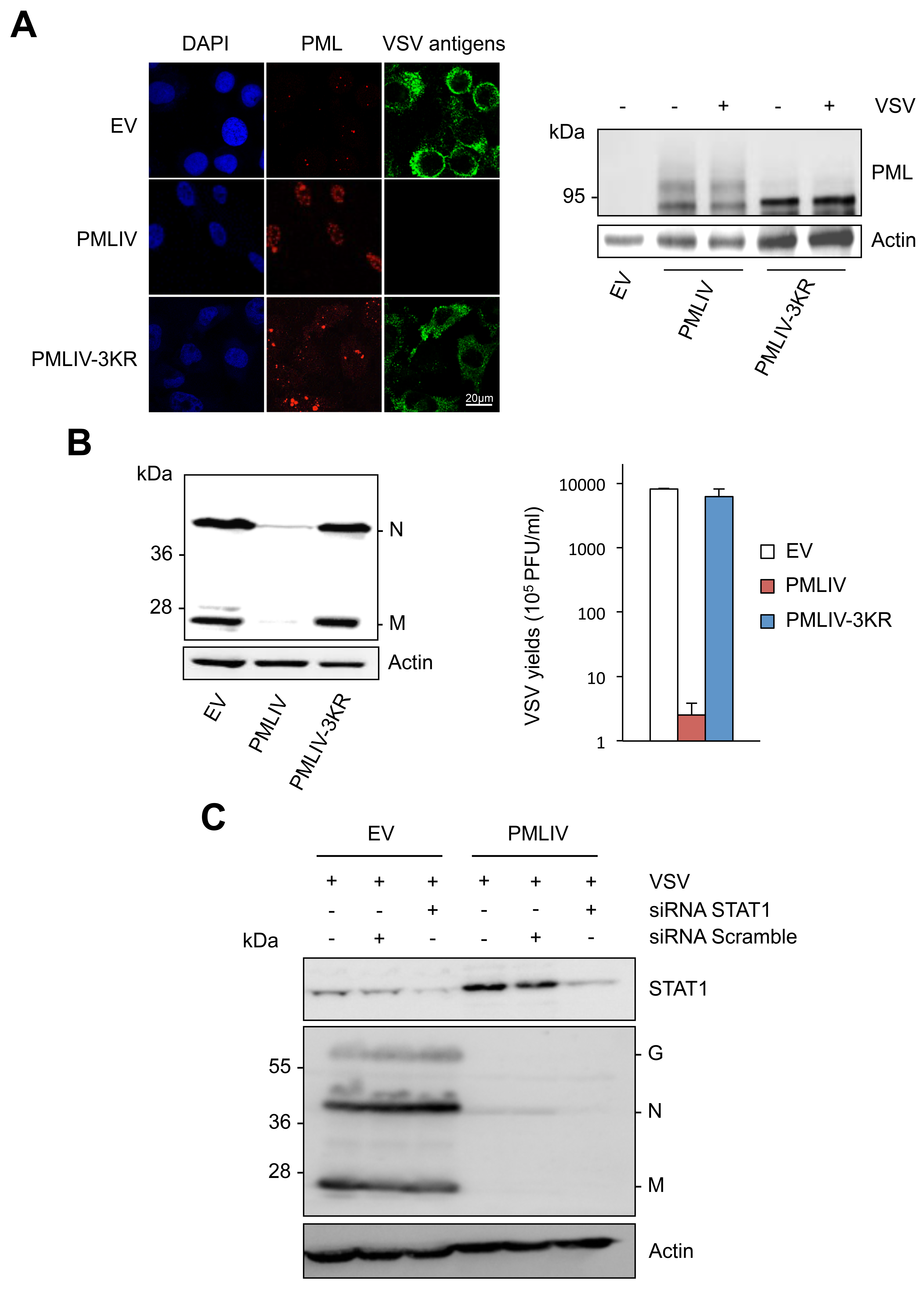

Supplement: Figure S2 — PMLIV SUMOylation is required for antiviral property. U373MG-EV, U373MG-PMLIV or U373MG-PMLIV-3KR cells were infected with VSV at an MOI of 1 for 8 h. (A/B) Double immunofluorescence staining was performed using monoclonal anti-PML (red) and rabbit anti-VSV (green) antibodies (A, left panel). Cell extracts were analyzed by Western blotting and revealed by antibodies directed against PML (A, right panel), VSV (B, left panel) or Actin. Supernatants from infected U373MG-EV, U373MG-PMLIV or U373MG-PMLIV-3KR cells were used for the determination of the virus yields (B, right panel). Means and standard deviations of two independent experiments are shown. (C) STAT1 depletion does not alter the intrinsic antiviral effect of PMLIV. U373MG-EV and U373MG-PMLIV cells were transfected with scramble (Sc) siRNA or STAT1-specific siRNA. Two days later, cells were infected with VSV at an MOI of 0.2 for 12 h. Cell extracts were used for the determination by Western blot of STAT1 and VSV protein expression. (TIF) [file ppat.1003975.s002.tif]
